# The *Pseudomonas syringae* pv. *tomato* Type III Effector HopM1 Suppresses Arabidopsis Defenses Independent of Suppressing Salicylic Acid Signaling and of Targeting AtMIN7

**DOI:** 10.1371/journal.pone.0082032

**Published:** 2013-12-06

**Authors:** Anju Gangadharan, Mysore-Venkatarau Sreerekha, Justin Whitehill, Jong Hyun Ham, David Mackey

**Affiliations:** 1 Department of Horticulture and Crop Science, The Ohio State University, Columbus, Ohio, United States of America; 2 Department of Molecular Genetics, The Ohio State University, Columbus, Ohio, United States of America; 3 Department of Plant Pathology, The Ohio State University, Columbus, Ohio, United States of America; University of the West of England, United Kingdom

## Abstract

*Pseudomonas syringae* pv tomato strain DC3000 (Pto) delivers several effector proteins promoting virulence, including HopM1, into plant cells via type III secretion. HopM1 contributes to full virulence of Pto by inducing degradation of Arabidopsis proteins, including AtMIN7, an ADP ribosylation factor-guanine nucleotide exchange factor. *Pseudomonas syringae* pv *phaseolicola* strain NPS3121 (Pph) lacks a functional HopM1 and elicits robust defenses in *Arabidopsis thaliana*, including accumulation of pathogenesis related 1 (PR-1) protein and deposition of callose-containing cell wall fortifications. We have examined the effects of heterologously expressed HopM1_Pto_ on Pph-induced defenses. HopM1 suppresses Pph-induced *PR-1* expression, a widely used marker for salicylic acid (SA) signaling and systemic acquired resistance. Surprisingly, HopM1 reduces *PR-1* expression without affecting SA accumulation and also suppresses the low levels of *PR-1* expression apparent in SA-signaling deficient plants. Further, HopM1 enhances the growth of Pto in SA-signaling deficient plants. AtMIN7 contributes to Pph-induced *PR-1* expression. However, HopM1 fails to degrade AtMIN7 during Pph infection and suppresses Pph-induced *PR-1* expression and callose deposition in wild-type and *atmin7* plants. We also show that the HopM1-mediated suppression of *PR-1* expression is not observed in plants lacking the TGA transcription factor, TGA3. Our data indicate that HopM1 promotes bacterial virulence independent of suppressing SA-signaling and links TGA3, AtMIN7, and other HopM1 targets to pathways distinct from the canonical SA-signaling pathway contributing to *PR-1* expression and callose deposition. Thus, efforts to understand this key effector must consider multiple targets and unexpected outputs of its action.

## Introduction

Microbes induce active plant defense responses, the elicitors of which can be divided into two classes, microbe/pathogen associated molecular patterns (MAMPs) and pathogen encoded effector proteins [1]. MAMPs are conserved microbial molecules that are directly recognized via plant-encoded MAMP-receptors [2]. Effectors are pathogen-encoded molecules that perturb host processes to promote pathogen virulence, but are sometimes recognized by plant-encoded resistance (R) proteins. Defense responses induced upon activation of MAMP-receptors and R-proteins are similar with the latter typically being more rapid and robust and more frequently associated with host cell death called the hypersensitive response (HR) [3].

Salicylic acid (SA) is one of several plant hormones produced during plant defense responses. In addition to its role in systemic resistance, SA-signaling also figures prominently in local MAMP- and effector-triggered defense responses [4]–[6]. SA accumulation re-localizes nonexpresser of *PR* genes (NPR1) to the nucleus where it functions as transcriptional co-activator [7]. SA regulates the activity and stability of NPR1 by binding to and affecting the interaction of NPR1 with two of its paralogues, NPR3 and NPR4 [8]. NPR1 activates the expression of genes involved in a defense-associated protein secretion pathway through TL1 promoter elements [9]. NPR1 also activates the expression of numerous *pathogenesis related* (*PR*) genes, including *PR-1*, by promoting the binding of TGA-family transcription factors to *as-1* promoter elements [10]. Out of 10 TGA transcription factors in Arabidopsis, 7 have been found to interact with NPR1 [11].

The *PR-1* promoter is also subject to additional layers of regulation. Multiple *PR-1* promoter elements are differentially regulated by positively and negatively acting WRKY and TGA transcription factors [12], [13]. Characterization of single and multiple knockout mutants revealed that TGA3 plays a significant role as an activator of *PR-1* expression [14]. ARR2, a key transcription factor in the cytokinin signaling pathway also binds to TGA3 and activates *PR-1* expression in a cytokinin-dependent manner [15]. Suppressor of NPR1 inducible1 (SNI1) negatively regulates *PR-1* expression, and that negative regulation is alleviated through interaction with DNA repair proteins [16]–[18]. Conversely, ethylene has been shown to enhance *PR-1* expression in the presence of low levels of SA [19].

Gram-negative phytopathogenic bacteria deliver type III effectors (T3Es) via a type III secretion system (TTSS) into the cytosol of plant cells. A major function of T3Es is to dampen MAMP- and/or effector-elicited defenses [20]. In *P. syringae*, the TTSS is encoded by a single cluster of genes adjacent to which is the conserved effector locus (CEL) [21]. The T3Es encoded in the CEL of *P. syringae* pv. tomato strain DC3000 (Pto) are necessary for full virulence. Compared to wild-type Pto, a mutant strain in which the CEL is deleted (PtoΔCEL) grows less and causes reduced disease symptoms in Arabidopsis and tomato [22], [23]. Plasmid expression of either of two T3Es from the CEL, HopM1 or AvrE1, can complement growth of PtoΔCEL and suppress SA-dependent deposition of callose in Arabidopsis [23]. Both effectors also contribute to Pto-induced necrosis on tomato [22], [24]. Therefore, HopM1 and AvrE1 are T3Es that make critical and functionally redundant contributions to the virulence of Pto.

Both HopM1 and AvrE1 are hypothesized to suppress plant defenses by disrupting G-protein-mediated endomembrane trafficking which is essential to plant defense [25]. Polarized secretion delivers antimicrobial cargo, including PR-proteins, reactive oxygen, and components or enzymes required for reinforcing the plant cell wall [9], [26]–[28]. During Pto infection, HopM1 causes the proteasome-dependent degradation of AtMIN7, an Arabidopsis ADP ribosylation factor-guanine nucleotide exchange factor (ARF-GEF) [29]. Thus, HopM1 likely alters the function of small G-proteins through elimination of a GEF protein. Degradation of AtMIN7 by HopM1 is blocked by immune responses activated through R-protein-mediated recognition of T3Es, AvrRpt2, AvrPphB and HopA1, indicating that part of the R-protein-mediated response might be to maintain the integrity of AtMIN7-dependent basal defense [30]. AtMIN7 (also known as BEN1) localizes to the *trans*-Golgi network/early endosome where it regulates endocytic cycling of plasma membrane localized proteins [30], [31]. Though no molecular function has been identified for AvrE1, mutations in motifs of AvrE1 that are putatively involved in GEF-mimicry disrupt its virulence contribution to Pto [32]–[34].

*P. syringae* pv. *phaseolicola* NPS3121 (Pph) is a pathogen of bean, but is non-pathogenic on Arabidopsis. We showed previously that Pph elicits robust defenses in Arabidopsis, including PR-1 protein accumulation and callose deposition, without eliciting host cell death [35]. A stop codon located midway through the *hopM1_Pph_* gene renders it nonfunctional [22], [29]. When Pph delivers HopM1_Pto_ (hereafter simply referred to as HopM1), Pph-induced defense responses in Arabidopsis are suppressed [35]. This strain, Pph (HopM1), elicits less PR-1 protein and callose than Pph, but still grows poorly on wild-type Arabidopsis. However, Pph (HopM1) does grow to high levels in Arabidopsis plants with mutations in defense signaling genes [35]. Thus, Pph/Arabidopsis is a useful model system to study a heterologous T3E delivered naturally through the TTSS of Pph.

Here we used Pph to investigate suppression of Arabidopsis defense responses by HopM1. Based on the putative role of AtMIN7 as a GEF, we hypothesized that HopM1 interferes with trafficking of PR-1 to the apoplast. However, HopM1 did not affect the ratio of secreted to non-secreted PR-1. Instead, HopM1 suppressed Pph-induced *PR-1* expression without reducing levels of SA. Pph-induced PR-1 protein accumulation was reduced in plants lacking TGA3 in addition to TGAs 2,5 and 6 and HopM1 could not further inhibit PR-1 accumulation in these plants, indicating that TGA3 is a positive regulator of the SA-independent pathway. Consistent with a virulence function of HopM1 that is independent of suppressing SA-signaling, HopM1 enhanced the growth of PtoΔCEL in SA-signaling deficient mutants. Surprisingly, HopM1 did not cause degradation of AtMIN7 during Pph infection and HopM1 suppressed Pph-induced callose deposition equally well in wild-type and *atmin7* mutant plants. Thus, we favor a model in which HopM1 suppresses plant defense independent of SA-signaling by targeting proteins other than AtMIN7.

## Materials and Methods

### Plant Materials and Bacterial Strains

*Arabidopsis thaliana* accession Col-0 was the wild type and the background for all mutants. The *atmin7* knock out (SALK_013761) plants, described previously [29], were obtained from ABRC at The Ohio State University, Columbus, Ohio. The RNA-null status of *atmin7* was confirmed by RT-PCR (data not shown). The *sid2-1*, *npr1-1*, *tga2-1 tga5-1 tga6-1*(*tga256*), *and tga2-1 tga3-1 tga5-1 tga6-1* (*tga2356*) mutants were reported previously [14], [36]–[38]. Plants used in the study were approximately five weeks old and were grown in cycles of 8 hours of light (115 µmol m^−2^ s^−1^) at 23°C and 16 hours of dark at 16°C.

The Pph strain used in the study was *P. syringae* pv. *phaseolicola* strain NPS 3121. Pph (HopM1) refers to strain 3121 carrying a plasmid expressing the type III effector HopM1 from *Pseudomonas syringae* pv. *tomato* strain DC3000, which was generated as described previously [35]. Bacteria were grown on KB plates with appropriate antibiotic selection, suspended in 10 mM MgCl_2_ and pressure infiltrated into the underside of leaves from a needleless 1 cm^3^ syringe at a concentration of 10^8^ CFU/ml (OD_600_ = 0.2) for *PR-1* expression, SA quantification and callose deposition assays or 10^6^ CFU/ml (OD_600_ = 0.002) for growth curve assays, which were performed as described [39]. Growth curve assays with Pto strains were done using an initial bacterial titer of 10^5^ CFU/ml (OD_600_ = 0.0002).

### Protein

Leaf protein preparations were made as previously described [40]. Samples were resolved on 12% SDS-PAGE gels (Mini-PROTEAN, Bio-Rad) and transferred to polyvinylidene difluoride membrane (Millipore, http://www.millipore.com/). Immunoblots were performed by standard procedures using anti-AtMIN7 sera at a dilution of 1∶3000 [29], anti-PR-1 sera at a dilution of 1∶5000, or anti-CSD1 sera at a dilution of 1∶500 [41]. The blots were developed using ECL Plus Western Blotting Detection Kit, images were acquired using a Storm 840 phosphorimager, and the protein bands were quantified using imageQuant software (GE Lifesciences, http://www.gelifesciences.com/).

### Isolation of Intercellular Fluid

To extract intercellular fluid, 8 plant leaves were collected and submerged completely in extraction buffer (300 mM NaCl, 50 mM NaPO_4_, pH 7.0) and the buffer was vacuum infiltrated until leaves were almost entirely water-soaked. The vacuum infiltrated leaves were then placed inside the barrel of a 10 ml needleless syringe from which the plunger was removed. The syringe was held inside a 50 ml centrifuge tube and spun for 20 minutes at 2000×g. Apoplastic fraction was retrieved from the bottom of the tube and the tissue remaining after the extraction of apoplastic fluid was used for cellular fraction. Protein separation by SDS-PAGE gel and immunoblotting were performed as described above.

### Quantitative Real Time PCR

*PR-1* transcript levels were measured using quantitative real time PCR (qRT-PCR). Total RNA was prepared from approximately 100 mg of plant tissue using the Qiagen Plant RNeasy mini prep kit (Qiagen, http://www.qiagen.com/). Total RNA was quantified using a nanodrop (NanoDrop™ ND-2000, http://www.nanodrop.com) and agarose gel electrophoresis, DNase treated with DNase1, Amplification Grade (Invitrogen, http://www.invitrogen.com/), and then cDNA synthesis was performed using Reverse Transcription system (Promega, http://www.promega.com/). Actin 2 was used as the reference gene for qRT-PCR. cDNA was amplified using the primer sets,

Actin 2 (at3g18780): fwd- 5′-ctaagctctcaagatcaaaggctta-3′
rev- 5′-ttaacattgcaaagagtttcaaggt-3′
PR-1 (at2g14610): fwd- 5′-ctacgcagaacaactaagaggcaac-3′
rev- 5′-ttggcacatccgagtctcactg-3′

For each biological replicate, each cDNA sample was tested in triplicates with each primer set. qRT-PCR reactions were set up using iQ SYBR green supermix and run in an iQ5 real-time PCR detection system (Bio-Rad).

### Callose Deposition Assay

Whole leaves were collected at approximately 16 hours after infiltration with bacterial suspension or buffer (10 mM MgCl_2_). Leaves were stained with aniline blue as previously described [39] and callose deposition was examined with a Nikon Eclipse 80i epifluorescence microscope (Nikon, http://www.nikon.com). The size and number of callose deposits were calculated using image J software.

### SA extraction and Quantification

Infiltrated leaves were ground in liquid N_2_ and 0.2 g was extracted twice overnight in 350 µl of 100% MeOH in the dark at 4°C. The supernatants were removed after centrifugation at 13,000 rpm for 10 minutes, pooled, and stored at −20°C until HPLC analyses. HPLC fluorescence analyses were performed using an Alliance 2690 separation module (Waters, www.waters.com/) equipped with an autosampler and a 474 Fluorescence Detector (Waters). The autosampler and column temperatures were set to 4 and 30°C, respectively. Chromatographic separation of methanolic extracts was carried out using a Waters Xterra™ RP18 analytical column (3.9 µm) coupled with a 3.0×20 mm guard column. The binary mobile phase consisted of water/acetic acid (A) (98∶2, v/v) and methanol/acetic acid (B) (98∶2, v/v) with a flow rate of 1 ml/min. The gradient was as follows (percentages refer to proportions of eluant B): 0 to 10% (0 to 4 min); 10 to 48% (4 to20 min); 48 to 100% (20 to38 min). The injection volume for all samples was 15 µl. Quantification of SA was achieved using fluorescence detection set to λ_em_ = 400 nm. Identification of SA was done by matching chromatographic profiles of individual samples to an external standard of SA. Individual peak areas of SA were quantified against an external standard of SA.

## Results

### HopM1 suppresses *PR-1* transcript accumulation independent of SA accumulation

**Figure 1:**
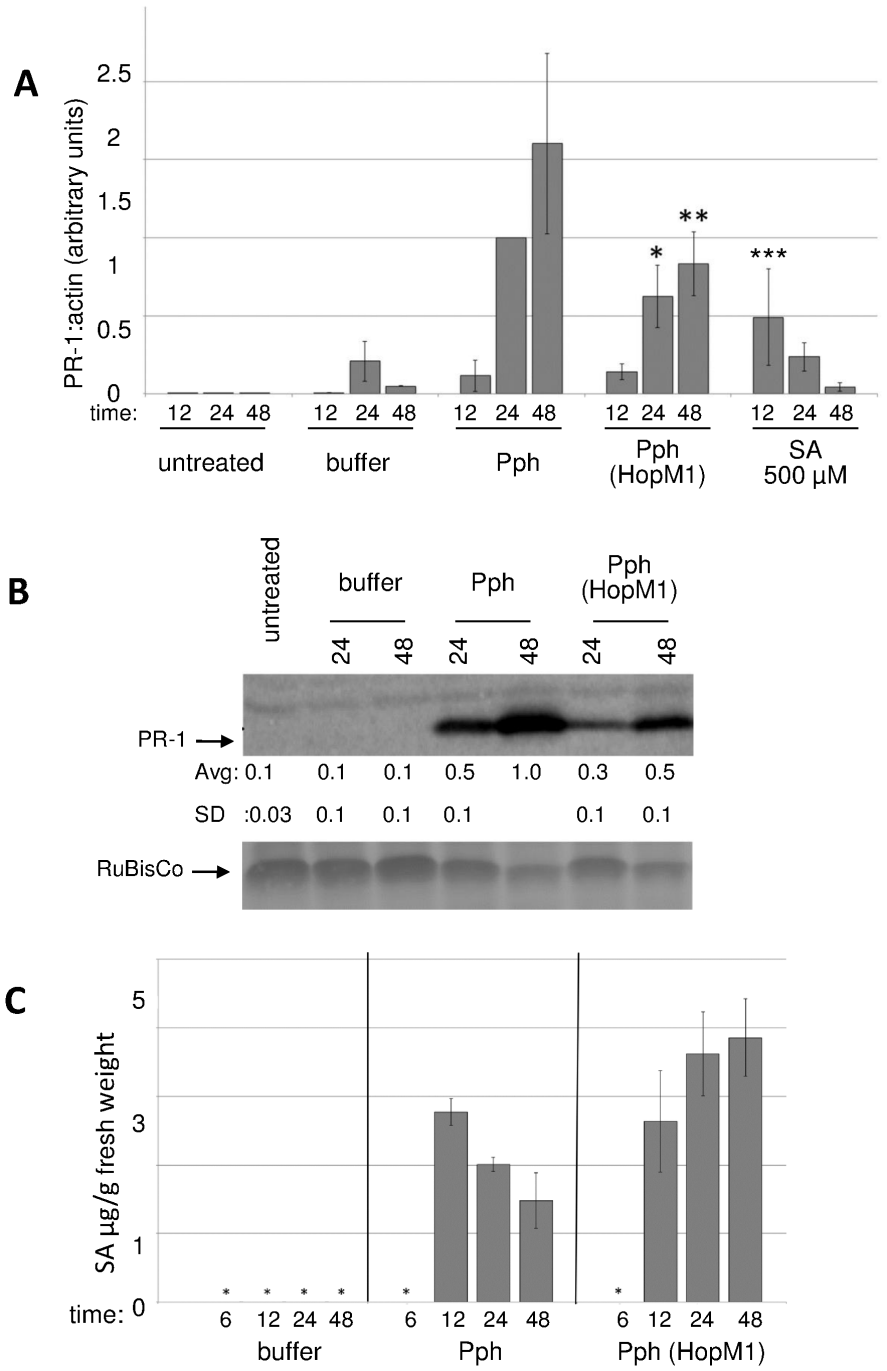
HopM1 suppresses Pph-induced *PR-1* expression without reducing accumulation of SA. A) Col-0 leaves were infiltrated with buffer, Pph, or Pph (HopM1), sprayed with 500 µM SA, or left untreated. Quantitative real time PCR (qRT-PCR) was used to measure the amount of *PR-1* transcript (relative to actin) at 12, 24, and 48 hai. The graph shows combined values from three independent biological replicates normalized with Pph in Col-0 at 24 hai set to 1. Error bars represent standard deviations. Paired two-tailed t-tests indicate that Pph (HopM1) induced less *PR-1* transcript than Pph at 24 (*, P = 0.0001) and 48 (**, P = 0.004) hai. *PR-1* accumulation with SA treatment was also significantly higher compared to Pph infiltration at 12 hai (***, P = 0.02). B) From leaves treated as in (A), total protein was extracted at 24 and 48 hai and subjected to anti-PR-1 immunoblotting. PR-1 protein in each sample was quantified and normalized with the value for Pph at 48 hai set to 1. The numbers shown below the blots indicate the average and standard deviation of combined data from three independent biological replicates. Paired two-tailed t-tests indicate that Pph (HopM1) induce less PR-1 protein than Pph at both 24 and 48 hai (P≤0.05). The cross-reacting band above PR-1 and ponceau staining of RuBisCo indicate equal loading of samples. C) From leaves treated as in (A), SA was extracted at 6, 12, 24, and 48 hai and measured by HPLC. Shown is the combined data from three biological replicates and error bars represent standard deviations. Samples marked with asterisks (*) contained too little SA for accurate quantification.

**Figure 2:**
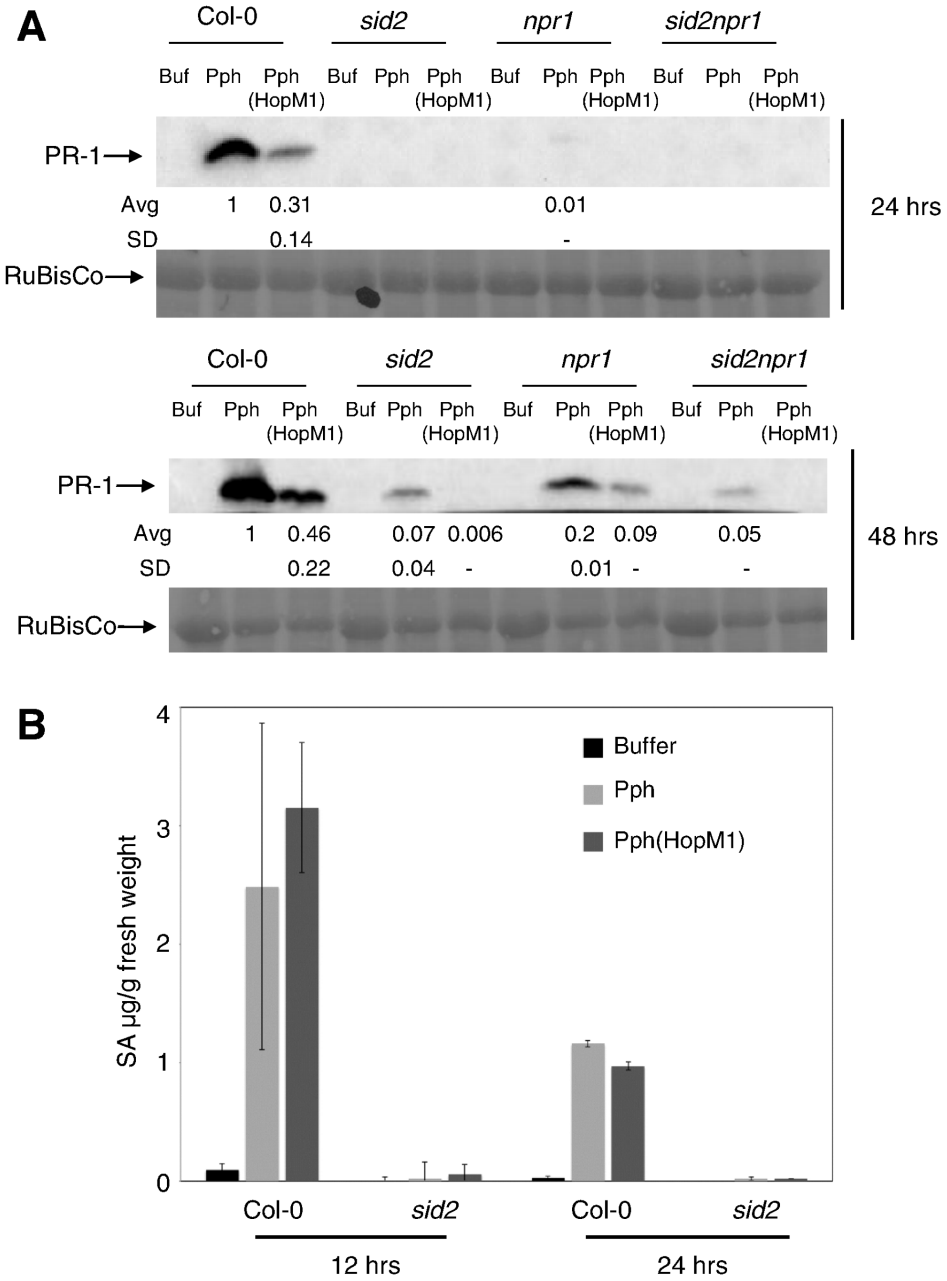
HopM1 suppresses Pph-induced *PR-1* expression in SA-signaling deficient backgrounds. A) Col-0 and SA-signaling mutant plants were infiltrated with buffer, Pph, or Pph (HopM1) and the amount of PR-1 protein was measured by immunoblotting at 24 and 48 hai. Quantified data were normalized with the amount of PR-1 induced by Pph in Col-0 set to 1. The average and standard deviation of values from three independent biological replicates are shown below the PR-1 blot. SD values for those samples that showed detectable PR-1 levels in only one replicate are not calculated (-). Ponceau stains of the membranes demonstrating equal protein loading are shown below. B) Plants were treated as in (A) and SA levels in leaves were measured at 12 and 24 hai. Shown is the combined data from three independent biological replicates and error bars represent standard deviations.

**Figure 3:**
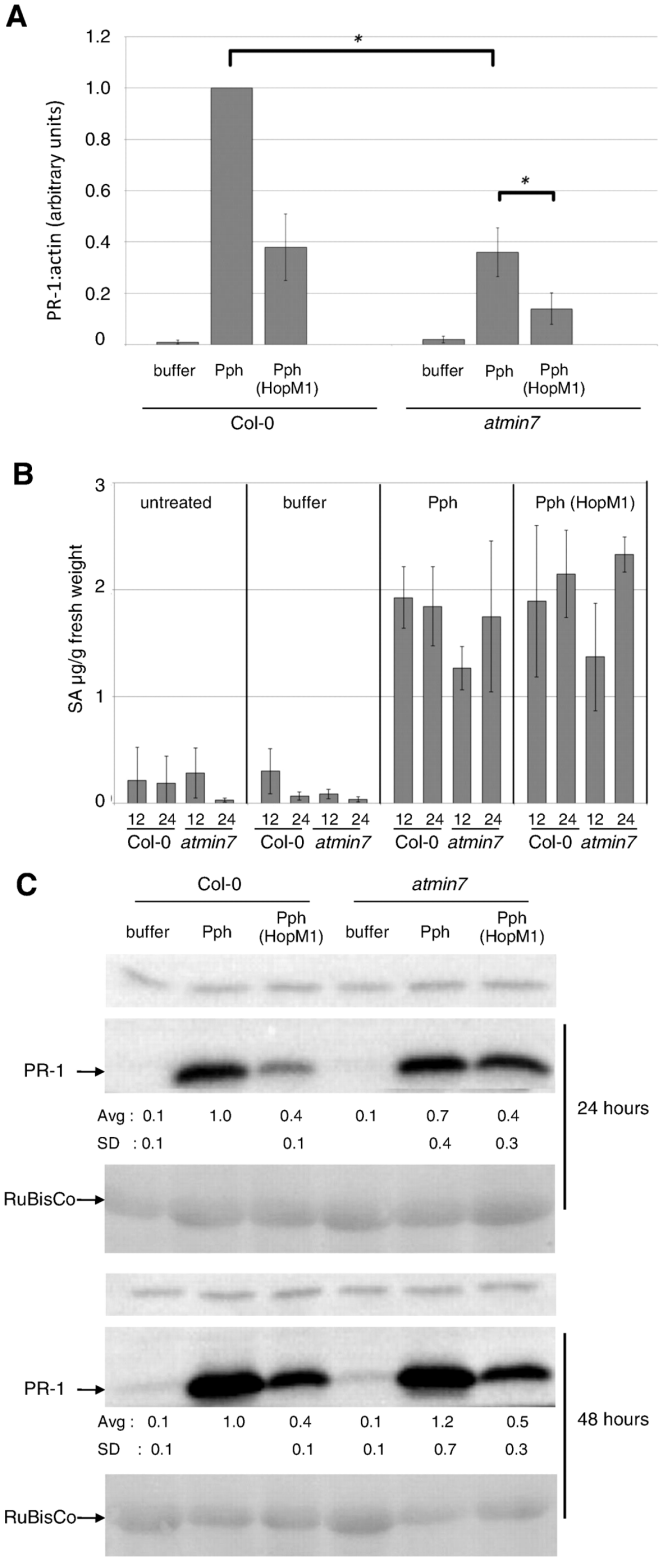
AtMIN7 positively regulates Pph-induced *PR-1* transcript accumulation without affecting accumulation of SA. A) Col-0 or *atmin7* plants were infiltrated with buffer, Pph, or Pph (HopM1). *PR-1* transcript levels were measured by qRT-PCR at 28 hai. The graph shows combined data from three independent biological replicates normalized with Pph in Col-0 set to 1. Paired two-tailed t-tests indicate significant differences between *PR-1* transcript levels induced by Pph in Col-0 versus *atmin7* and by Pph versus Pph (HopM1) in *atmin7* plants (*, P<0.001). B) Plants were treated as in (A) and SA levels in leaves were measured at 12 and 24 hai. Shown is the combined data from three independent biological replicates and error bars represent standard deviations. Paired two-tailed t-tests indicate that SA levels induced by Pph in Col-0 versus *atmin7* differed at 12 hai (P = 0.03) and that differences with Pph versus Pph (HopM1) in Col-0 or *atmin7* were not apparent at 12 or 24 hai (P≥0.5). C) Plants were treated as in (A) and total protein samples from 24 and 48 hai were subjected to anti-PR-1 immunoblotting. Quantified data were normalized with the amount of PR-1 protein induced by Pph in Col-0 set to 1. The average and standard deviation values for four independent biological replicates are shown below the blots. Paired two-tailed t-tests did not show significant differences between PR-1 levels in Col-0 versus *atmin7* plants infiltrated with Pph or Pph (HopM1) (P≥0.3). The cross-reacting band above PR-1 and ponceau staining of RuBisCo indicate equal loading of samples.

We first hypothesized that HopM1 suppresses secretion of defense associated cargo, including PR-1 protein. This hypothesis was based on (1) the targeting of an ARF-GEF by HopM1 [29] and (2) the fact that efficient secretion of PR-1 protein is necessary for effective resistance [9], [28]. Since a reduction in the secretion efficiency of PR-1 protein is not apparent when Pph expresses HopM1 (Fig. S1), this hypothesis is not supported. However, since reduced PR-1 secretion might be offset by reduced stability of non-secreted PR-1, we cannot rule out an effect of HopM1 on secretion of PR-1 or other defense cargo. These results prompted us to look for other effects of HopM1 on *PR-1* expression. The levels of *PR-1* transcript following challenge of Arabidopsis with Pph or Pph (HopM1) were measured with quantitative real-time PCR (qRT-PCR) (Fig. 1A). The induced expression of *PR-1* was only ∼60%, and 50% as much in Pph (HopM1)-infected leaves as in Pph-infected leaves at 24 and 48 hours after infiltration (hai), respectively. These reductions in transcript were similar to the observed reductions in PR-1 protein accumulation (Fig. 1B). We previously showed that *SID2*, which often is required for defense-associated SA production, is required for full Pph-induced PR-1 protein accumulation [35], [38]. Thus, we speculated that HopM1 might reduce Pph-induced *PR-1* transcript accumulation by suppressing accumulation of SA. To test this idea, levels of free SA in leaves of Arabidopsis infiltrated with buffer, Pph, or Pph (HopM1) were measured (Fig. 1C). Very low levels of SA were present at 6 hai with Pph or Pph (HopM1) and at all tested time points following infiltration with buffer. Infiltration with Pph or Pph (HopM1) elicited accumulation of free SA at 12, 24, and 48 hours and the levels of SA were not reduced by expression of HopM1. The apparent increase in SA induced by Pph (HopM1) relative to Pph at 24 and 48 hai observed in figure 1C was not reproducible (e.g. see 24 hour samples in figs. 2B and 3B). Altogether, figure 1A–C shows that HopM1 suppresses the Pph-induced accumulation of *PR-1* transcripts and protein downstream or independent of SA accumulation.

To further examine the relationship between HopM1 and SA-signaling, we tested the effect of exogenously applied SA on PR-1 protein accumulation after infiltration with Pph (HopM1). We reasoned that if HopM1 functioned independent of SA-signaling, then the reduced levels of PR-1 induced by Pph (HopM1) would not be restored by SA-supplementation. Col-0 leaves were infiltrated with buffer, Pph, or Pph (HopM1). After the infiltrate dried, plants were sprayed with 300 µM SA or were left unsprayed. *PR-1* expression was measured by anti-PR-1 immunoblotting at 24 and 48 hai (Fig. S2). At each time point, the amount of PR-1 protein induced by infiltration with Pph (HopM1) was set to 1. Notably, the level of PR-1 accumulation induced by Pph was not significantly increased by SA spray at either time point, indicating that SA-signaling is fully activated by Pph. Thus, the observation that the lower level of PR-1 induced by Pph (HopM1) was not restored by spraying with supplemental SA indicates that SA is still not rate limiting and therefore HopM1 likely targets an SA-independent process. This result reinforces the conclusion that HopM1 can suppress *PR-1* expression in a manner that is independent of the level of SA.

We also examined the ability of HopM1 to suppress *PR-1* expression in plants with compromised SA-accumulation and/or signaling. Col-0, *sid2*, *npr1*, or *sid2npr1* plants were infiltrated with Pph or Pph (HopM1) and the accumulation of PR-1 protein was measured at 24 and 48 hai (Fig. 2A). At each time point, the amount of PR-1 protein induced by infiltration of Col-0 with Pph was set to 1. Pph-induced PR-1 accumulation was reduced in each of the mutants. HopM1 further suppressed the low levels of PR-1 that accumulated by 48 hai in the mutants. Because plants can produce SA from chorismate (dependent on *SID2*) or from phenylalanine (independent of *SID2*), we sought to determine whether Pph elicits SA accumulation in the *sid2* mutant. SA measurements in *sid2* plants indicated that infiltration with buffer, Pph, or Pph (HopM1) each elicit similar, low levels of SA (Fig. 2B). These data provide evidence for the existence of an SA-independent pathway for *PR-1* expression and indicate that HopM1 affects *PR-1* expression at least in part by suppressing the SA-independent defense pathway.

### AtMIN7 positively contributes to *PR-1* expression induced by Pph

The ability of HopM1 to eliminate AtMIN7 is part of its contribution to full growth of the Arabidopsis pathogen Pto [29]. We compared Col-0 and *atmin7* mutant plants to test the contribution of AtMIN7 to *PR-1* expression in response to Pph (Fig. 3A). At 28 hai, Pph elicited only ∼40% as much *PR-1* transcript in *atmin7* as in Col-0, indicating a positive contribution of AtMIN7 to this Pph-induced defense response.

HopM1 has been shown to interact with and target proteins other than AtMIN7 (Nomura *et al.*, 2006). Consistent with figure 1A, Pph (HopM1) induced accumulation of less *PR-1* transcript than did Pph in Col-0. Figure 3A shows that HopM1 similarly reduced *PR-1* transcript accumulation in the absence of AtMIN7. Following infiltration of *atmin7* plants, Pph (HopM1) induced only ∼40% as much *PR-1* transcript as did Pph. The combined effects of the absence of AtMIN7 and the action of HopM1 are striking; Pph (HopM1) in *atmin7* induced only ∼15% as much *PR-1* transcript accumulation as did Pph in Col-0. Since HopM1 reduces *PR-1* transcript levels induced by Pph to a similar extent in Col-0 and *atmin7* plants, this defense suppressing activity is mediated independent of targeting AtMIN7 or through elimination of AtMIN7 and perturbation of additional plant targets.

We used *atmin7* plants to further examine the role of SA-independent *PR-1* expression in response to Pph infection. SA levels were measured at 12 and 24 hai of *atmin7* and Col-0 plants with buffer, Pph, or Pph (HopM1) (Fig. 3B). As was observed in figure 1C, the accumulation of SA in wild-type Arabidopsis in response to Pph was not reduced by the expression of HopM1. Furthermore, the levels of Pph- and Pph (HopM1)-induced SA were similar in wild-type and *atmin7* mutant plants. Although levels of *PR-1* transcript were lowest following infiltration of *atmin7* with Pph (HopM1), the amount of free SA was still not reduced. These results provide additional evidence that HopM1 suppresses *PR-1* expression and AtMIN7 makes a positive contribution towards the elicitation of this defense response without affecting SA accumulation.

### AtMIN7 contributes to *PR-1* transcript accumulation following infiltration of Pph but not spraying of SA

**Figure 4:**
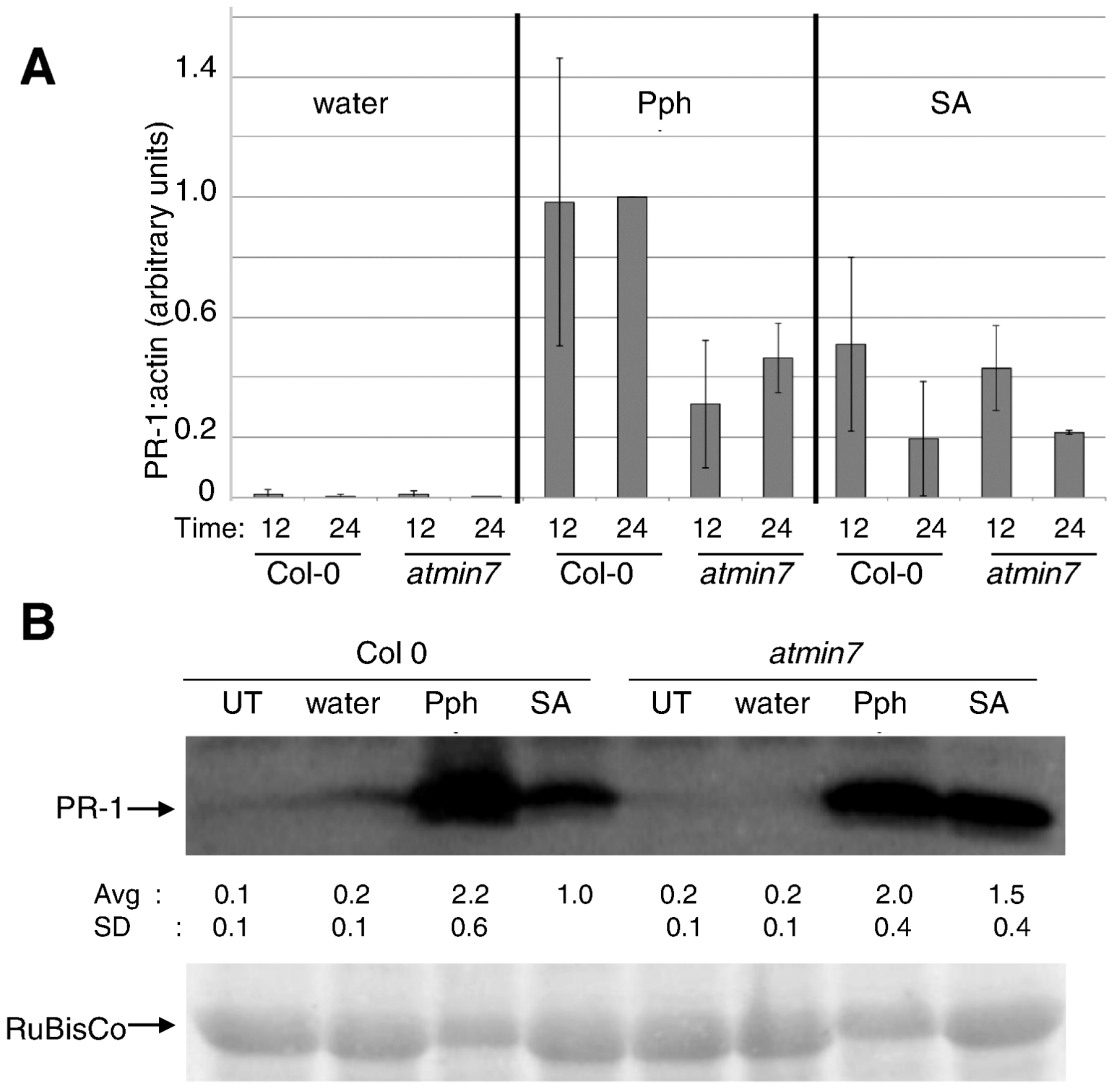
The positive contribution of AtMIN7 to Pph-induced *PR-1* transcript accumulation is independent of SA-signaling. A) Col-0 or *atmin7* plants were sprayed with water, infiltrated with Pph, or sprayed with 300 µM SA. *PR-1* transcript levels were measured by qRT-PCR at 12 and 24 hours after spray or infiltration. Samples were normalized with the value for Pph in Col-0 at 24 hai set to 1 and the averages and standard deviations of data from four independent biological replicates is shown. Paired two-tailed t-tests indicate that transcript levels induced by SA spray did not vary significantly in Col-0 versus *atmin7* at 12 or 24 hai (P≥0.7). B) Plants were treated as in (A) and 48 hours after spray or infiltration total protein from treated leaves was subjected to anti-PR-1 immunoblotting. Quantified data were normalized with the amount of PR-1 protein induced by SA in Col-0 set to 1. Average and standard deviations from three independent biological replicates are shown below the blot. The cross-reacting band above PR-1 and ponceau staining of RuBisCo indicate equal loading of samples.

AtMIN7 could contribute to Pph-induced *PR-1* expression in either of two ways: (1) it could be required for SA-signaling downstream from SA accumulation (*e.g.* regulating the nuclear function of NPR1) or (2) it could otherwise regulate *PR-1* expression (*e.g.* a Pph-induced pathway that independently impinges on the *PR-1* promoter or that alters *PR-1* transcript stability). A prediction based on the second hypothesis is that the absence of AtMIN7 will not affect *PR-1* expression induced by direct application of SA. To test this hypothesis, Col-0 and *atmin7* plants were sprayed with water or 300 µM SA or infiltrated with Pph and the levels of *PR-1* transcript were measured after 12 and 24 hours (Fig. 4A). As observed in figure 3, Pph infiltration elicited ∼40% as much *PR-1* transcript in *atmin7* as in Col-0. Contrary to Pph infiltration, spraying with SA elicited comparable levels of *PR-1* transcript in *atmin7* and Col-0 plants. These results demonstrate that AtMIN7 is not required for SA-induced *PR-1* transcript accumulation. Thus, we infer that AtMIN7 contributes to Pph-induced *PR-1* transcript accumulation independent of the canonical SA-signaling pathway.

### Contrary to its positive contribution to *PR-1* transcript accumulation, AtMIN7 negatively affects PR-1 protein accumulation

During the course of this work we repeatedly observed that, on a per *PR-1* transcript basis, the PR-1 protein accumulates more efficiently in *atmin7* than in Col-0 plants. For example, the levels of PR-1 protein induced by Pph infiltration were similar between Col-0 and *atmin7* plants (Fig. 3C and 4B), despite the lower levels of *PR-1* transcript in *atmin7* (Fig. 3A and 4A). Figure 4 also shows that the enhanced PR-1 protein accumulation in *atmin7* is not specific to Pph infiltration. Spraying Col-0 and *atmin7* plants with SA-induced comparable levels of *PR-1* transcript in both genotypes, but induced more PR-1 protein in *atmin7*. Collectively, these results indicate that the absence of AtMIN7 reduces Pph-induced *PR-1* transcript accumulation while nonetheless increasing the amount of PR-1 protein that accumulates per transcript. The same effect is not observed when Pph expresses HopM1, presumably because HopM1 fails to eliminate AtMIN7 during Pph infection (see below).

### Target(s) of HopM1 other than AtMIN7 are critical for Pph-induced defense responses

**Figure 5:**
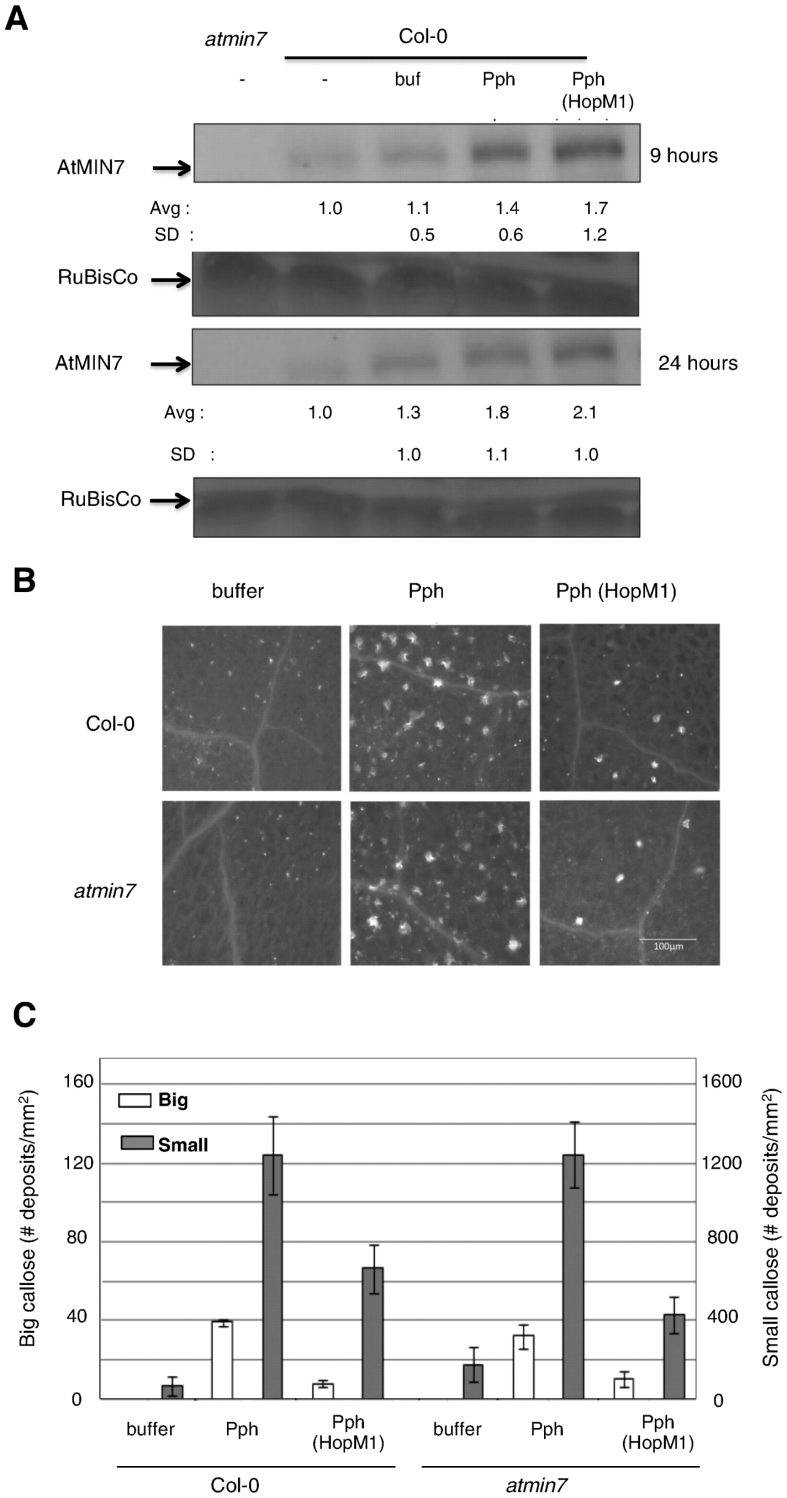
Targets of HopM1 other than AtMIN7 are critical to Pph-induced defense responses. A) Col-0 plants were infiltrated with buffer, Pph, Pph (HopM1) or Col-0 and *atmin7* plants were left untreated. AtMIN7 protein levels were measured from samples collected at 9 and 24 hai by immunoblotting. Quantified data were normalized with the amount of AtMIN7 protein present in untreated Col-0 at each time point set to 1.The average and standard deviations from multiple replicates are shown below the blots. Ponceau staining of RuBisCo indicate equal loading. B) Col-0 or *atmin7* plants were infiltrated with buffer, Pph, or Pph (HopM1). After 16 hours, leaves were cleared and stained with aniline blue and visualized by fluorescent microscopy. Representative pictures are shown. The scale bar in the bottom right picture is 100 microns. C) Image J was used to count small and big callose deposits. Shown are the average and standard deviations from three independent biological replicates. Paired two-tailed t-tests indicate that the callose deposits induced in Col-0 versus *atmin7* did not differ for Pph or Pph (HopM1) (P>0.6).

Suppression of *PR-1* transcript levels in *atmin7* plants by HopM1 raised the possibility of HopM1 functioning either independent of AtMIN7 or through perturbation of other targets in addition to AtMIN7. Recent studies have shown that ETI can suppress HopM1 mediated degradation of AtMIN7 [30]. Pph elicits much stronger defense responses, including *PR-1* expression, than does a TTSS-deficient mutant of Pph, indicating that Pph may elicit ETI in Arabidopsis [35]. Thus we tested for degradation of AtMIN7 by HopM1 during Pph infection by examining levels of AtMIN7 protein after challenge of Col-0 plants with Pph or Pph (HopM1). At 9 and 24 hai, Pph induced elevated levels of AtMIN7 protein and HopM1 failed to eliminate protein accumulation (Fig. 5A). Since HopM1 fails to eliminate AtMIN7 during a Pph infection, we concluded that *PR-1* expression is suppressed via targets other than AtMIN7.

We also wished to compare the effect of HopM1 on Pph-induced *PR-1* expression with its ability to suppress Pph-induced callose deposition. We showed previously that Pph induces two distinct types of callose deposits in Col-0 that are classified as small and big callose [35]. Small callose deposits are sub-cellular and indistinguishable from those elicited by flg22 or TTSS-deficient bacteria, while big callose deposits are entire mesophyll cells encased in callose. We also showed that the numbers of small and big callose deposits induced by Pph are comparable in wild-type Col-0 and in the *sid2* and *npr1* mutants [35]. Thus, SA-signaling is not required for Pph-induced callose deposition. We wondered if HopM1 suppresses Pph-induced callose deposition via the same mechanism that it suppresses Pph-induced *PR-1* transcript accumulation.

To address this question, we compared the pattern of callose deposition in Col-0 and *atmin7* following infiltration with Pph or Pph (HopM1). Because callose deposition in response to PtoΔCEL is reduced in *atmin7* [29], we expected the mutation would also impair Pph-induced callose deposition. We reproduced the results of Nomura *et al.* with PtoΔCEL (data not shown), but surprisingly Pph elicited indistinguishable patterns of callose deposition in Col-0 and *atmin7* plants (Fig. 5B,C). Thus, AtMIN7 is not necessary for the callose response against Pph. Furthermore, the ability of HopM1 to suppress Pph-induced callose is similar in Col-0 and *atmin7* plants (Fig. 5B,C). Thus, HopM1 suppresses Pph-induced callose through a mechanism independent of AtMIN7. We are unable to ascertain if AtMIN7 contributes in a functionally redundant manner with one or more additional targets of HopM1. However, contrary to its positive contribution to Pph-induced *PR-1* transcript accumulation, AtMIN7 does not make a detectable contribution to Pph-induced callose deposition. These results, which indicate (1) the existence of an AtMIN7-independent pathway for Pph-induced callose deposition and (2) HopM1-mediated suppression of this pathway through targets other than AtMIN7, highlight the complexity of the plant defense signaling network and that the network is targeted in multiple ways by HopM1.

### TGA3 transcription factor is a positive regulator of Pph-induced, SA-independent pathway for *PR-1* expression

**Figure 6:**
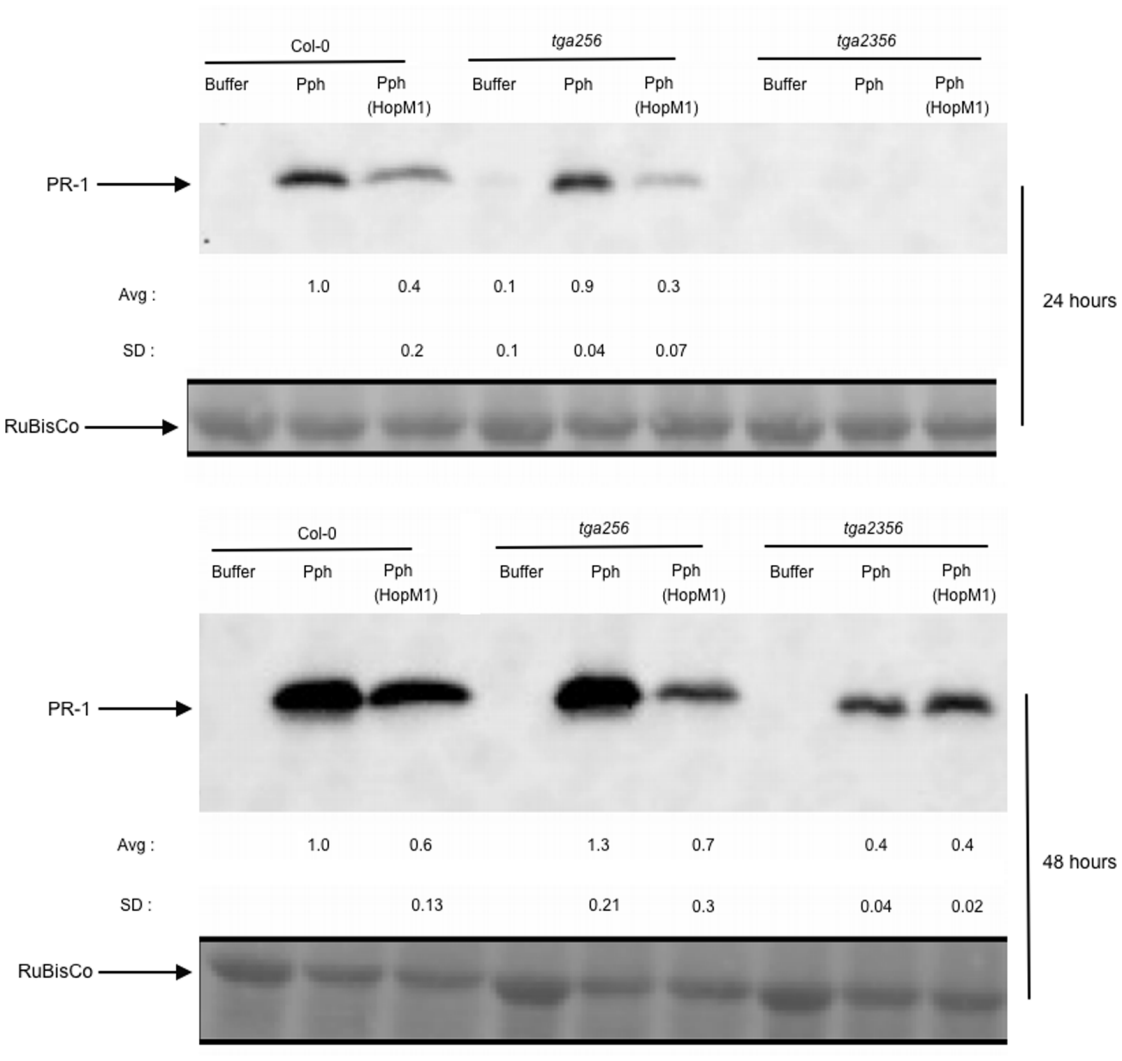
TGA3 is a positive regulator of Pph-induced PR-1 protein accumulation. Col-0, *tga256*, and *tga2356* plants were infiltrated with buffer, Pph or Pph(HopM1) and PR-1 protein levels were measured from samples collected at 24 and 48hai by immunoblotting. Protein levels were quantified and the data were normalized with amount of protein induced by Pph at each time point set to 1. The average and standard deviations from multiple replicates are shown below the blots. Ponceau staining of RuBisCo indicate equal loading.

Members of the TGA family of transcription factors are known to be required for SA-dependent *PR-1* expression. TGA triple (*tga256*) and quadruple (*tga2356*) mutants are defective in SA-induced *PR-1* expression [14], [36]. We checked if TGA family members are involved in SA-independent PR-1 protein accumulation. Wild-type,*tga256* and *tga2356* plants were inoculated with Pph or Pph (HopM1) and PR-1 protein accumulation was analyzed at 24 and 48 hai (Fig. 6). As already noted, PR-1 protein levels were reduced after inoculation with Pph (HopM1) relative to Pph in wild-type plants. In *tga256* mutants, contrary to the inability of SA to induce *PR-1* expression, Pph induced accumulation of PR-1 protein. In this background, HopM1 suppressed PR-1 accumulation. In the quadruple *tga2356* mutant, Pph induced low levels of PR-1 protein compared to either wild-type or the *tga256* triple mutant. Remarkably, HopM1 was unable to suppress PR-1 accumulation in *tga2356* plants indicating that TGA3 plays a significant role in Pph-induced PR-1 protein accumulation, possibly through its involvement in both SA-dependent as well as SA-independent pathways.

### HopM1 suppresses defense responses independent of SA-signaling to promote virulence in Pto

Ham et al showed that HopM1 enhances the growth of Pph only when multiple defense pathways are interrupted [35]. Our comparison of the growth levels of Pph and Pph (HopM1) in wild type and SA-signaling mutant plants supported this finding. Pph or Pph (HopM1) each failed to proliferate in Col-0 or SA-signaling mutants (Fig S3).

**Figure 7:**
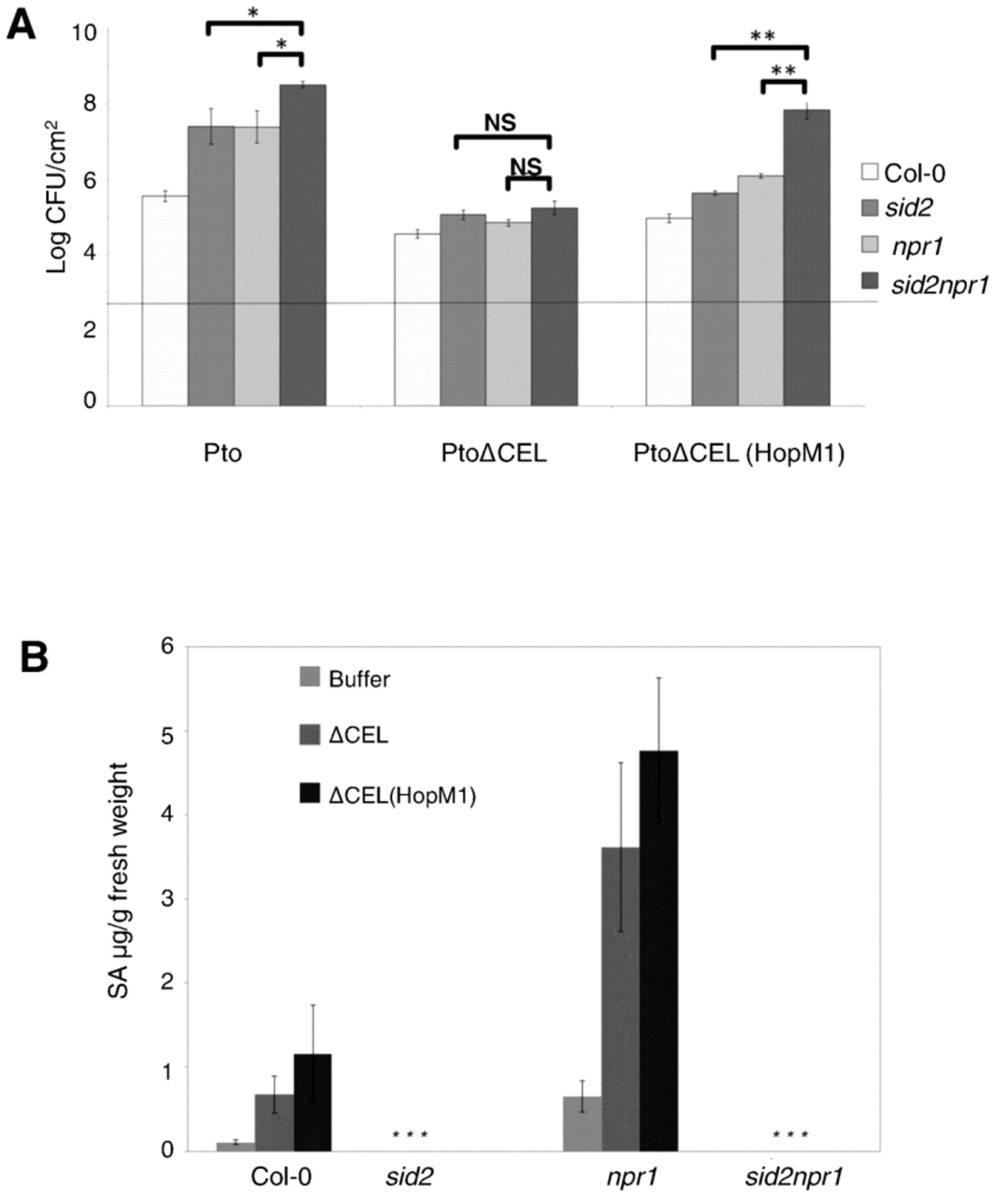
HopM1 suppresses SA independent defense responses to promote bacterial virulence. A) Col-0 and SA-signaling deficient plants were infiltrated with Pto, PtoΔCEL and PtoΔCEL (HopM1) at a concentration of 10^5^ CFU/ml. Growth of bacteria was assessed at 4 days after infiltration. Shown is the combined data and standard deviations from 3 independent biological replicates. The dashed line represents bacterial levels at day 0. Paired two-tailed t-tests were used to compare the growth of individual strains in *sid2npr1* versus *sid2* or *npr1* (ns, not significant; *, P≤0.05; **, P<0.0001). B) Plants were infiltrated with buffer, PtoΔCEL or PtoΔCEL (HopM1) and SA levels in leaves were measured at 15 hai. Shown is the combined data from three independent biological replicates and error bars represent standard deviations. Asterisks (*) indicate that SA levels were below the limit of detection (0.05 µg of SA/g fresh weight).

To examine the ability of HopM1 to promote bacterial growth while avoiding the use of multiple mutant plants, we turned to the ΔCEL mutant of Pto. Col-0 and SA-signaling mutant plants were infiltrated with Pto, PtoΔCEL, or PtoΔCEL (HopM1) and bacterial growth was compared after 4 days. In *sid2* and *npr1* single mutants, the growth of PtoΔCEL was partially complemented by HopM1 showing that the suppression of defenses in addition to those induced by SA are important for virulence promotion by HopM1(Fig. 7A). Interestingly, PtoΔCEL (HopM1) grew as well as wild type Pto in *sid2npr1* double mutants, strongly supporting our conclusion that HopM1 promotes virulence independent of suppressing SA-mediated defenses. Why HopM1 promotes the growth of PtoΔCEL better in the double mutant than in the single mutants is not known.

To support our assertion that HopM1 functions independent of suppressing SA-signaling, we checked free SA levels in wild type and mutant plants following infiltration with these bacterial strains. As expected, SA did not accumulate to detectable levels in *sid2* and *sid2npr1* mutants (Fig. 7B). Also, *npr1* plants accumulated more SA compared to Col-0 (Fig. 7B), consistent with the earlier findings that NPR1 mediates negative feedback regulation on the production of SA [42]. Thus, HopM1 promotes bacterial virulence independent of suppressing SA-signaling.

## Discussion

*PR-1* expression is a widely monitored readout of SA-signaling in plant defense. Indeed, SA contributes significantly to Pph-induced expression of *PR-1*. However, Pph also elicits an SA-independent pathway that contributes to *PR-1* expression. Pph elicits significant accumulation of PR-1 protein without eliciting SA accumulation in *sid2* mutant plants (Fig. 2). Highlighting the potential significance of this non-canonical pathway to plant defense against *P. syringae*, HopM1, a key T3E from the CEL, suppresses SA-independent expression of *PR-1* induced by Pph through perturbation of one or more of its targets in Arabidopsis (Fig. S4).

Our initial hypothesis that HopM1 destabilizes Pph-induced PR-1 protein by suppressing its trafficking is now disfavored for two reasons. First, our analysis of PR-1 secretion efficiency did not reveal an effect of HopM1 on PR-1 trafficking (Fig. S1). However, it remained possible that HopM1 suppresses PR-1 secretion and non-secreted PR-1 is destabilized producing a negligible net effect on the ratio of extracellular to intracellular PR-1. Inconsistent with this idea, PR-1 protein appears to be stabilized in *atmin7* mutant plants. Similar to its hypothesized role in trafficking of auxin transporters [31], AtMIN7 may promote trafficking of PR-1 through the early endosome to the vacuole where it is unstable. Second, our observation that HopM1 suppresses Pph-induced *PR-1* transcript accumulation to levels that correlate well with the suppression of PR-1 protein accumulation provides a more parsimonious explanation.

There are several possible explanations why the secretion efficiency of PR-1 protein in SA-sprayed plants was higher than in leaves infiltrated with Pph or Pph (HopM1) (Fig. S1). Pph, although non-pathogenic on Arabidopsis, may be somewhat effective at inhibiting defense associated secretion of PR-1. Or, incorporation of PR-1 into cell wall thickenings associated with the defense response against non-pathogenic bacteria [28] may reduce the efficiency with which PR-1 protein is extracted into the apoplastic fluid. Or, SA spray may induce the expression of genes required for efficient secretion of PR-1 into the apoplast more potently than Pph infiltration [9]. Regardless of the cause, this observation demonstrates that extraction of PR-1 protein in the apoplastic fluid can be highly efficient, lending more weight to the similar and low extraction efficiencies observed following challenge with Pph or Pph (HopM1).

Our data support the hypothesis that HopM1 suppresses an SA-independent pathway and that TGA3, AtMIN7, and other targets of HopM1 positively regulate the pathway (Fig. S4). Pph elicits less *PR-1* transcript accumulation when it expresses HopM1 or infects *atmin7* plants (Fig. 3A). Similar to the effect of HopM1, the *atmin7* mutant shows reduced *PR-1* expression without a reduction in SA accumulation (Fig. 3B) and exogenous SA application elicits comparable levels of *PR-1* transcript accumulation in Col-0 and *atmin7* (Fig. 4A), indicating that AtMIN7 positively regulates the SA-independent pathway. However, in our growth conditions, HopM1 fails to eliminate AtMIN7 during Pph infection suggesting that it is affecting the SA-independent pathway by targeting proteins other than AtMIN7 (Fig. 5A). It will be of great interest to determine the nature of the HopM1-suppressed pathway and what genes, in addition to *PR-1*, are regulated by it.

TGA transcription factors are key regulators of SA-dependent and SA-independent *PR-1* expression [44]. We show here that TGA3 is a positive regulator of Pph-induced *PR-1* expression (Fig. 6). The dependence of TGA3 is observed in the background of the *tga256* triple mutant, which surprisingly has no effect on Pph-induced *PR-1* expression. Without testing the effectiveness of HopM1 in a *tga2356* quadruple mutant that is also deficient in SA-signaling, we cannot say for sure that the contribution of TGA3 is mediated in an SA-independent manner. However, regulation of *PR-1* expression by TGA3 has been shown to be not completely dependent on NPR1, a key regulator of SA-signaling pathway [14], [44]. TGA3 is known to interact with the cytokinin reponse regulator, ARR2 and this interaction is reported to be required for cytokinin dependent *PR-1* activation [15]. ARR2 is also shown to be involved in ethylene signaling and is observed to affect the expression of genes involved in other hormone signaling pathways as well as biotic and abiotic stress responses [45]. Recently it has been reported that ARR2 is degraded in a proteasome dependent manner in order to maintain cytokinin signaling output at optimal levels for plant growth and development [46]. By targeting a regulator of TGA3 function, such as ARR2, HopM1 might be able to disrupt multiple defense signaling pathways.

HopM1 also suppresses callose deposition via multiple plant targets. HopM1 suppresses SA-dependent callose deposition elicited by PtoΔCEL [23] and elimination of AtMIN7 contributes to this activity since PtoΔCEL elicits reduced callose in the *atmin7* mutant [29]. The SA-dependence of callose deposition by PtoΔCEL is due to the ability of coronatine to inhibit an SA-independent pathway supporting this cell wall response [43]. HopM1 also suppresses the deposition of SA-independent callose elicited by Pph [35]. Remarkably, the *atmin7* mutation has no effect on Pph-induced callose deposition or its suppression by HopM1 (Figs. 5B and 5C). Since HopM1 suppresses *PR-1* expression and callose deposition by elimination of targets other than AtMIN7, Pph-induced defenses will be useful readouts to study the role of HopM1 targets other than AtMIN7. Notably, the ability of HopM1 to suppress SA-independent responses is biologically relevant as it significantly increases the growth of PtoΔCEL in plants deficient in SA-signaling.

## Supporting Information

Figure S1 **HopM1 does not alter the secretion efficiency of PR-1 protein following Pph infiltration.** A) Col-0 plants were infiltrated with buffer, Pph, or Pph (HopM1) or were sprayed with 500 µM SA. After 24 or 48 hours, apoplastic fluid was extracted from treated leaves and total protein was prepared from leaves after apoplastic fluid extraction (Cellular fraction). Apoplastic and cellular fractions were subjected to anti-PR-1 immunoblotting (middle panels, lower band). Ponceau staining of RuBisCo and immunoblot detection of CSD1, a cytosolic protein, and the anti-PR-1 cross-reacting band above PR-1 indicate that non-secreted cellular proteins are efficiently retained in the cellular sample. The reduced amount of RuBisCo in the bacterial infiltrated samples correlates with observed tissue collapse. B) For four separate biological replicates of the experiment shown in figure 1A, PR-1 protein in each sample was quantified and the ratios of apoplastic to cellular for each treatment were determined. Within each experiment at 24 and 48 hours, the ratios were normalized with the Pph treatment set to 1. The graph shows the composite of the normalized data from the four experiments and the error bars represent standard deviations. Paired two tail t-tests indicate that the apoplastic:cellular ratios of samples sprayed with SA differed significantly from the samples infiltrated with either Pph or Pph (HopM1) at 48 hours (*, P = 0.03 for both comparisons). C) To establish a standard curve for PR-1 quantification, a protein extract with very high levels of PR-1 was serially diluted into an extract with no detectable PR-1 and subjected to anti-PR-1 immunoblotting. The graph at right shows the relationship between band quantification and amount of PR-1 protein. PR-1 was similarly quantified in other figures within the paper.
(TIF)

Figure S2 **HopM1 suppresses PR-1 accumulation independent of SA.** Col-0 plants were challenged in two stages. First, leaves were infiltrated with buffer, Pph, or Pph (HopM1). Second, after 2 hours (sufficient time for the infiltrated tissue to dry) the plants were left untreated or were sprayed with 300 µM SA, indicated as (−) or (+), respectively. At 24 and 48 hours after the infiltration step, total protein was subjected to anti-PR-1 immunoblotting. Quantified data was normalized for each time point with the amount of PR-1 induced by Pph (HopM1) set to 1. The average and standard deviation values from five biological replicates (except for Pph infiltration followed by SA spray, which was from three biological replicates) are shown below the representative blots. Paired two-tailed t-tests indicate that protein levels induced by unsprayed, Pph or Pph (HopM1)-infiltrated leaves did not significantly differ from Pph or Pph (HopM1)-infiltrated leaves subsequently sprayed with SA at 24 (P≥0.3) or 48 hours (P≥0.4). Ponceau stains of the membranes demonstrate equal protein loading.
(TIFF)

Figure S3 **HopM1 fails to promote growth of Pph in SA signaling mutants.** Col-0, *sid2, npr1* and *sid2npr1* plants were infiltrated with 10^6^ CFU/ml of either Pph or Pph (HopM1). Bacterial growth was assayed at 0, 2 and 4 days after infiltration. Graph represents the combined result from 5 different biological replicates for day 4 and 2 biological replicates for day 2. The dashed line represents bacterial levels at day 0. Error bars represent standard deviations.
(TIF)

Figure S4 **Model for suppression of Pph-induced** ***PR-1*** **expression and callose deposition by HopM1.** Pph elicits *PR-1* expression and callose deposition via pathways independent of the canonical SID2- and NPR1-dependent SA-signaling pathway. AtMIN7 and TGA3 positively regulate the alternate pathway leading to *PR-1* expression. HopM1 inhibits both the alternate pathways by eliminating targets other than AtMIN7.
(TIF)

## References

[1] Chisholm ST, Coaker G, Day B, Staskawicz BJ (2006) Host-microbe interactions: shaping the evolution of the plant immune response. Cell 124: 803–814. 16497589 10.1016/j.cell.2006.02.008

[2] Mackey D, McFall AJ (2006) MAMPs and MIMPs: proposed classifications for inducers of innate immunity. Molecular microbiology 61: 1365–1371. 16899081 10.1111/j.1365-2958.2006.05311.x

[3] Jones JD, Dangl JL (2006) The plant immune system. Nature 444: 323–329. 17108957 10.1038/nature05286

[4] Clay NK, Adio AM, Denoux C, Jander G, Ausubel FM (2009) Glucosinolate metabolites required for an Arabidopsis innate immune response. Science 323: 95–101. 19095898 10.1126/science.1164627 PMC2630859

[5] Glazebrook J (2005) Contrasting mechanisms of defense against biotrophic and necrotrophic pathogens. Annu Rev Phytopathol 43: 205–227. 16078883 10.1146/annurev.phyto.43.040204.135923

[6] Tsuda K, Sato M, Glazebrook J, Cohen JD, Katagiri F (2008) Interplay between MAMP-triggered and SA-mediated defense responses. Plant J 53: 763–775. 18005228 10.1111/j.1365-313X.2007.03369.x

[7] Mou Z, Fan W, Dong X (2003) Inducers of plant systemic acquired resistance regulate NPR1 function through redox changes. Cell 113: 935–944. 12837250 10.1016/s0092-8674(03)00429-x

[8] Fu ZQ, Yan S, Saleh A, Wang W, Ruble J, et al (2012) NPR3 and NPR4 are receptors for the immune signal salicylic acid in plants. Nature 486: 228–232. 22699612 10.1038/nature11162 PMC3376392

[9] Wang D, Weaver ND, Kesarwani M, Dong X (2005) Induction of protein secretory pathway is required for systemic acquired resistance. Science 308: 1036–1040. 15890886 10.1126/science.1108791

[10] Fan W, Dong X (2002) In vivo interaction between NPR1 and transcription factor TGA2 leads to Salicylic acid-mediated gene activation in Arabidopsis. Plant Cell 14: 1377–1389. 12084833 10.1105/tpc.001628 PMC150786

[11] Jakoby M, Weisshaar B, Droge-Laser W, Vicente-Carbajosa J, Tiedemann J, et al (2002) bZIP transcription factors in Arabidopsis. Trends Plant Sci 7: 106–111. 11906833 10.1016/s1360-1385(01)02223-3

[12] Pape S, Thurow C, Gatz C (2010) The Arabidopsis PR-1 promoter contains multiple integration sites for the coactivator NPR1 and the repressor SNI1. Plant Physiol 154: 1805–1818. 20935179 10.1104/pp.110.165563 PMC2996008

[13] Lebel E, Heifetz P, Thorne L, Uknes S, Ryals J, et al (1998) Functional analysis of regulatory sequences controlling PR-1 gene expression in Arabidopsis. Plant J 16: 223–233. 9839467 10.1046/j.1365-313x.1998.00288.x

[14] Kesarwani M, Yoo J, Dong X (2007) Genetic interactions of TGA transcription factors in the regulation of pathogenesis-related genes and disease resistance in Arabidopsis. Plant Physiol 144: 336–346. 17369431 10.1104/pp.106.095299 PMC1913812

[15] Choi J, Huh SU, Kojima M, Sakakibara H, Paek KH, et al (2010) The cytokinin-activated transcription factor ARR2 promotes plant immunity via TGA3/NPR1-dependent salicylic acid signaling in Arabidopsis. Dev Cell 19: 284–295. 20708590 10.1016/j.devcel.2010.07.011

[16] Li X, Zhang Y, Clarke JD, Li Y, Dong X (1999) Identification and cloning of a negative regulator of systemic acquired resistance, *SNI1*, through a screen for suppressors of *npr1-1* . Cell 98: 329–339. 10458608 10.1016/s0092-8674(00)81962-5

[17] Mosher RA, Durrant WE, Wang D, Song J, Dong X (2006) A comprehensive structure-function analysis of Arabidopsis SNI1 defines essential regions and transcriptional repressor activity. Plant Cell 18: 1750–1765. 16766691 10.1105/tpc.105.039677 PMC1488919

[18] Song J, Durrant WE, Wang S, Yan S, Tan EH, et al (2011) DNA repair proteins are directly involved in regulation of gene expression during plant immune response. Cell Host Microbe 9: 115–124. 21320694 10.1016/j.chom.2011.01.011

[19] Lawton KA, Potter SL, Uknes S, Ryals J (1994) Acquired resistance signal transduction in Arabidopsis is ethylene independent. Plant Cell 6: 581–588. 12244251 10.1105/tpc.6.5.581 PMC160460

[20] Hann DR, Gimenez-Ibanez S, Rathjen JP (2010) Bacterial virulence effectors and their activities. Curr Opin Plant Biol 13: 388–393. 20466583 10.1016/j.pbi.2010.04.003

[21] Alfano JR, Charkowski AO, Deng WL, Badel JL, Petnicki-Ocwieja T, et al (2000) The Pseudomonas syringae Hrp pathogenicity island has a tripartite mosaic structure composed of a cluster of type III secretion genes bounded by exchangeable effector and conserved effector loci that contribute to parasitic fitness and pathogenicity in plants. Proc Natl Acad Sci U S A 97: 4856–4861. 10781092 10.1073/pnas.97.9.4856 PMC18322

[22] Badel JL, Shimizu R, Oh HS, Collmer A (2006) A Pseudomonas syringae pv. tomato avrE1/hopM1 mutant is severely reduced in growth and lesion formation in tomato. Mol Plant Microbe Interact 19: 99–111. 16529372 10.1094/MPMI-19-0099

[23] DebRoy S, Thilmony R, Kwack YB, Nomura K, He SY (2004) A family of conserved bacterial effectors inhibits salicylic acid-mediated basal immunity and promotes disease necrosis in plants. Proc Natl Acad Sci U S A 101: 9927–9932. 15210989 10.1073/pnas.0401601101 PMC470775

[24] Badel JL, Nomura K, Bandyopadhyay S, Shimizu R, Collmer A, et al (2003) Pseudomonas syringae pv. tomato DC3000 HopPtoM (CEL ORF3) is important for lesion formation but not growth in tomato and is secreted and translocated by the Hrp type III secretion system in a chaperone-dependent manner. Mol Microbiol 49: 1239–1251. 12940984 10.1046/j.1365-2958.2003.03647.x

[25] Bednarek P, Kwon C, Schulze-Lefert P (2010) Not a peripheral issue: secretion in plant-microbe interactions. Curr Opin Plant Biol 13: 378–387. 20558098 10.1016/j.pbi.2010.05.002

[26] Assaad FF, Qiu JL, Youngs H, Ehrhardt D, Zimmerli L, et al (2004) The PEN1 syntaxin defines a novel cellular compartment upon fungal attack and is required for the timely assembly of papillae. Mol Biol Cell 15: 5118–5129. 15342780 10.1091/mbc.E04-02-0140 PMC524786

[27] Collins NC, Thordal-Christensen H, Lipka V, Bau S, Kombrink E, et al (2003) SNARE-protein-mediated disease resistance at the plant cell wall. Nature 425: 973–977. 14586469 10.1038/nature02076

[28] Kalde M, Nuhse TS, Findlay K, Peck SC (2007) The syntaxin SYP132 contributes to plant resistance against bacteria and secretion of pathogenesis-related protein 1. Proc Natl Acad Sci U S A 104: 11850–11855. 17592123 10.1073/pnas.0701083104 PMC1913864

[29] Nomura K, Debroy S, Lee YH, Pumplin N, Jones J, et al (2006) A bacterial virulence protein suppresses host innate immunity to cause plant disease. Science 313: 220–223. 16840699 10.1126/science.1129523

[30] Nomura K, Mecey C, Lee YN, Imboden LA, Chang JH, et al (2011) Effector-triggered immunity blocks pathogen degradation of an immunity-associated vesicle traffic regulator in Arabidopsis. Proc Natl Acad Sci U S A 108: 10774–10779. 21670267 10.1073/pnas.1103338108 PMC3127868

[31] Tanaka H, Kitakura S, De Rycke R, De Groodt R, Friml J (2009) Fluorescence imaging-based screen identifies ARF GEF component of early endosomal trafficking. Curr Biol 19: 391–397. 19230664 10.1016/j.cub.2009.01.057

[32] Alto NM, Shao F, Lazar CS, Brost RL, Chua G, et al (2006) Identification of a bacterial type III effector family with G protein mimicry functions. Cell 124: 133–145. 16413487 10.1016/j.cell.2005.10.031

[33] Ham JH, Majerczak DR, Nomura K, Mecey C, Uribe F, et al (2009) Multiple Activities of the Plant Pathogen Type III Effector Proteins WtsE and AvrE Require WxxxE Motifs. Mol Plant Microbe Interact 22: 703–712. 19445595 10.1094/MPMI-22-6-0703 PMC2748107

[34] Huang Z, Sutton SE, Wallenfang AJ, Orchard RC, Wu X, et al (2009) Structural insights into host GTPase isoform selection by a family of bacterial GEF mimics. Nat Struct Mol Biol 16: 853–860. 19620963 10.1038/nsmb.1647 PMC5130228

[35] Ham JH, Kim MG, Lee SY, Mackey D (2007) Layered basal defenses underlie non-host resistance of Arabidopsis to Pseudomonas syringae pv. phaseolicola. Plant J 51: 604–616. 17573803 10.1111/j.1365-313X.2007.03165.x

[36] Zhang Y, Tessaro MJ, Lassner M, Li X (2003) Knockout analysis of Arabidopsis transcription factors TGA2, TGA5, and TGA6 reveals their redundant and essential roles in systemic acquired resistance. Plant Cell 15: 2647–2653. 14576289 10.1105/tpc.014894 PMC280568

[37] Cao H, Glazebrook J, Clarke JD, Volko S, Dong X (1997) The Arabidopsis NPR1 gene that controls systemic acquired resistance encodes a novel protein containing ankyrin repeats. Cell 88: 57–63. 9019406 10.1016/s0092-8674(00)81858-9

[38] Wildermuth MC, Dewdney J, Wu G, Ausubel FM (2001) Isochorismate synthase is required to synthesize salicylic acid for plant defence. Nature 414: 562–565. 11734859 10.1038/35107108

[39] Kim MG, Mackey D (2008) Measuring cell-wall-based defenses and their effect on bacterial growth in Arabidopsis. Methods Mol Biol 415: 443–452. 18370170 10.1007/978-1-59745-570-1_26

[40] Kim MG, da Cunha L, McFall AJ, Belkhadir Y, DebRoy S, et al (2005) Two Pseudomonas syringae type III effectors inhibit RIN4-regulated basal defense in Arabidopsis. Cell 121: 749–759. 15935761 10.1016/j.cell.2005.03.025

[41] Kliebenstein DJ, Dietrich RA, Martin AC, Last RL, Dangl JL (1999) LSD1 regulates Salicylic Acid induction of copper-zinc superoxide dismutase in *Arabidopsis thaliana* . Mol Plant Microbe Interact 12: 1022–1026. 10550898 10.1094/MPMI.1999.12.11.1022

[42] Shah J (2003) The salicylic acid loop in plant defense. Curr Opin Plant Biol 6: 365–371. 12873532 10.1016/s1369-5266(03)00058-x

[43] Geng X, Cheng J, Gangadharan A, Mackey D (2012) The coronatine toxin of Pseudomonas syringae is a multifunctional suppressor of Arabidopsis defense. Plant Cell 24: 4763–4774. 23204405 10.1105/tpc.112.105312 PMC3531865

[44] Gatz C (2013) From pioneers to team players: TGA transcription factors provide a molecular link between different stress pathways. Mol Plant Microbe Interact 26: 151–159. 23013435 10.1094/MPMI-04-12-0078-IA

[45] Hass C, Lohrmann J, Albrecht V, Sweere U, Hummel F, et al (2004) The response regulator 2 mediates ethylene signalling and hormone signal integration in Arabidopsis. EMBO J 23: 3290–3302. 15282545 10.1038/sj.emboj.7600337 PMC514511

[46] Kim K, Ryu H, Cho YH, Scacchi E, Sabatini S, et al (2012) Cytokinin-facilitated proteolysis of ARABIDOPSIS RESPONSE REGULATOR 2 attenuates signaling output in two-component circuitry. Plant J 69: 934–945. 22050482 10.1111/j.1365-313X.2011.04843.x

