# Supplementary figures and images for: The Pseudomonas syringae pv. tomato Type III Effector HopM1 Suppresses Arabidopsis Defenses Independent of Suppressing Salicylic Acid Signaling and of Targeting AtMIN7
Source: PLoS One. 2013 Dec 6;8(12):e82032. doi: 10.1371/journal.pone.0082032 (PMC3855835; doi:10.1371/journal.pone.0082032)

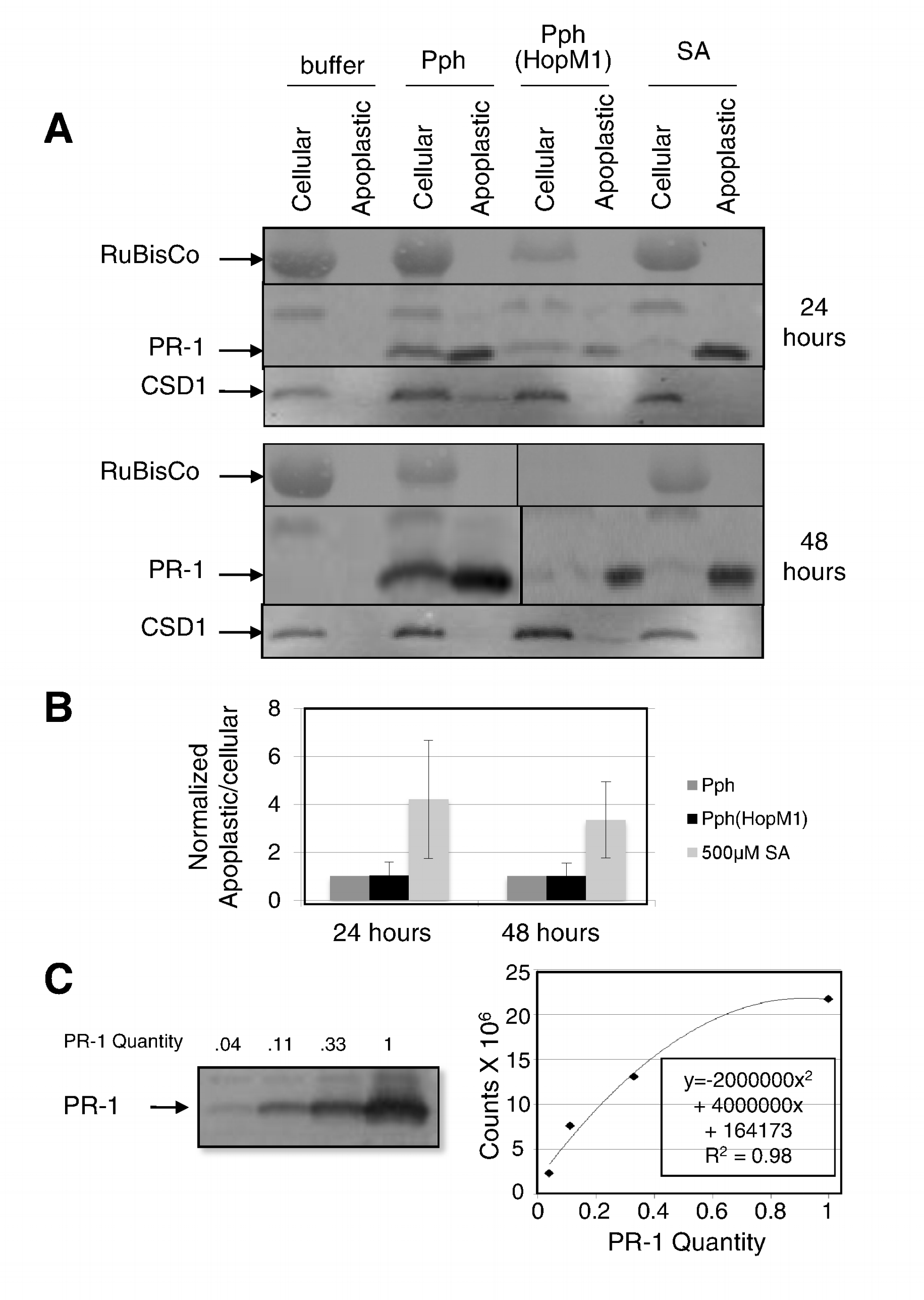

Supplement: Figure S1 — HopM1 does not alter the secretion efficiency of PR-1 protein following Pph infiltration. A) Col-0 plants were infiltrated with buffer, Pph, or Pph (HopM1) or were sprayed with 500 µM SA. After 24 or 48 hours, apoplastic fluid was extracted from treated leaves and total protein was prepared from leaves after apoplastic fluid extraction (Cellular fraction). Apoplastic and cellular fractions were subjected to anti-PR-1 immunoblotting (middle panels, lower band). Ponceau staining of RuBisCo and immunoblot detection of CSD1, a cytosolic protein, and the anti-PR-1 cross-reacting band above PR-1 indicate that non-secreted cellular proteins are efficiently retained in the cellular sample. The reduced amount of RuBisCo in the bacterial infiltrated samples correlates with observed tissue collapse. B) For four separate biological replicates of the experiment shown in figure 1A, PR-1 protein in each sample was quantified and the ratios of apoplastic to cellular for each treatment were determined. Within each experiment at 24 and 48 hours, the ratios were normalized with the Pph treatment set to 1. The graph shows the composite of the normalized data from the four experiments and the error bars represent standard deviations. Paired two tail t-tests indicate that the apoplastic:cellular ratios of samples sprayed with SA differed significantly from the samples infiltrated with either Pph or Pph (HopM1) at 48 hours (*, P = 0.03 for both comparisons). C) To establish a standard curve for PR-1 quantification, a protein extract with very high levels of PR-1 was serially diluted into an extract with no detectable PR-1 and subjected to anti-PR-1 immunoblotting. The graph at right shows the relationship between band quantification and amount of PR-1 protein. PR-1 was similarly quantified in other figures within the paper. (TIF) [file pone.0082032.s001.tif]

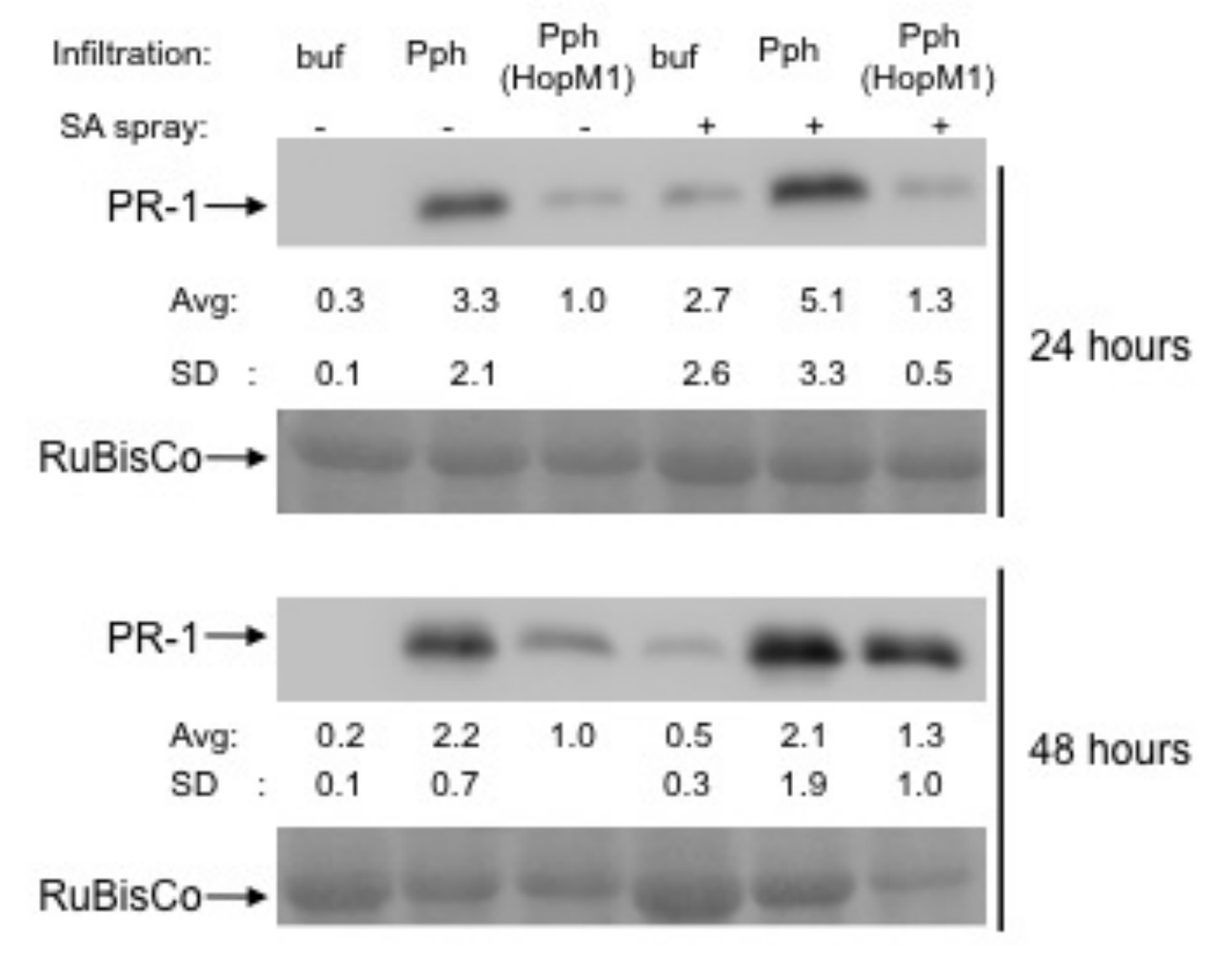

Supplement: Figure S2 — HopM1 suppresses PR-1 accumulation independent of SA. Col-0 plants were challenged in two stages. First, leaves were infiltrated with buffer, Pph, or Pph (HopM1). Second, after 2 hours (sufficient time for the infiltrated tissue to dry) the plants were left untreated or were sprayed with 300 µM SA, indicated as (−) or (+), respectively. At 24 and 48 hours after the infiltration step, total protein was subjected to anti-PR-1 immunoblotting. Quantified data was normalized for each time point with the amount of PR-1 induced by Pph (HopM1) set to 1. The average and standard deviation values from five biological replicates (except for Pph infiltration followed by SA spray, which was from three biological replicates) are shown below the representative blots. Paired two-tailed t-tests indicate that protein levels induced by unsprayed, Pph or Pph (HopM1)-infiltrated leaves did not significantly differ from Pph or Pph (HopM1)-infiltrated leaves subsequently sprayed with SA at 24 (P≥0.3) or 48 hours (P≥0.4). Ponceau stains of the membranes demonstrate equal protein loading. (TIFF) [file pone.0082032.s002.tiff]

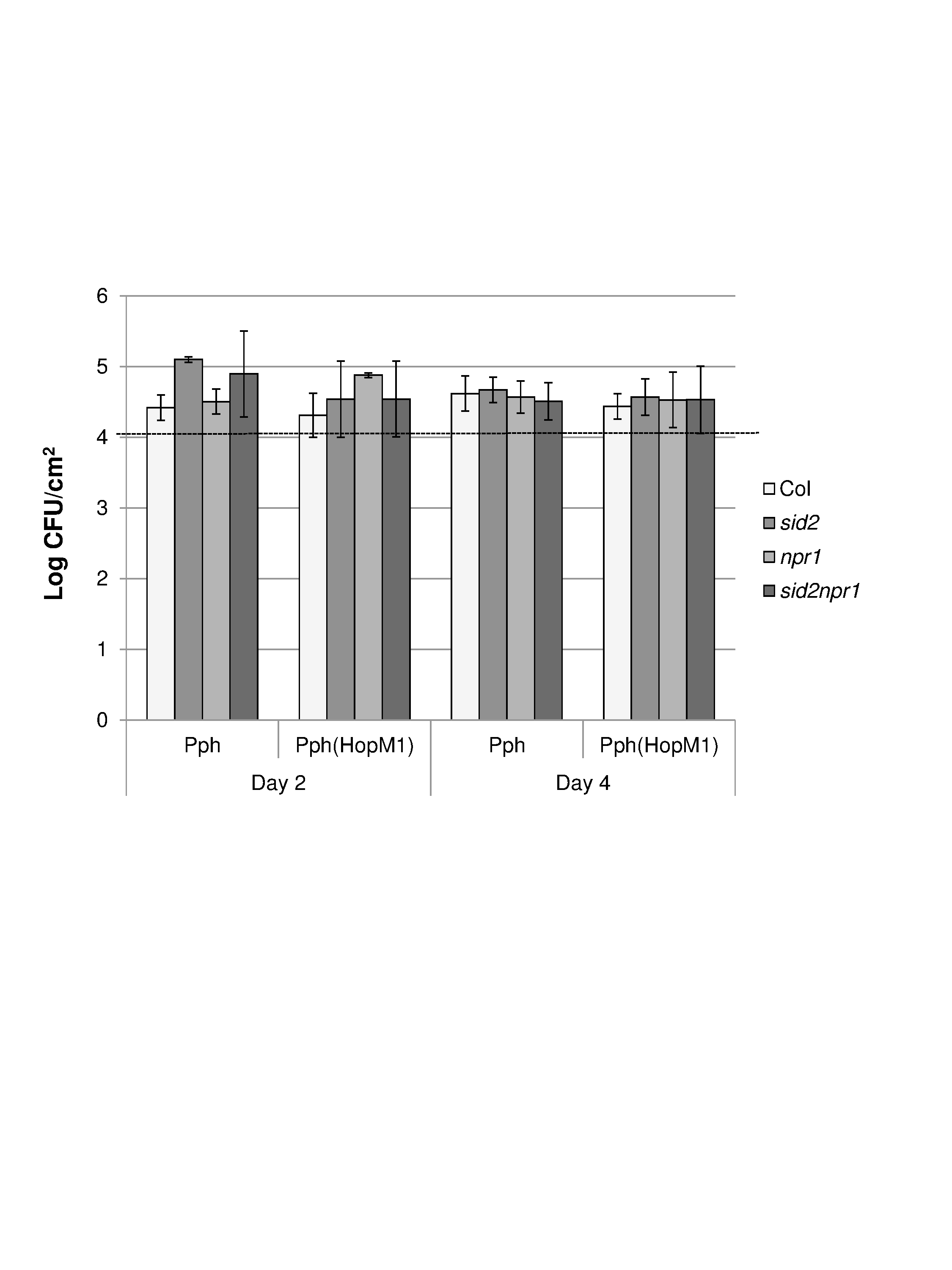

Supplement: Figure S3 — HopM1 fails to promote growth of Pph in SA signaling mutants. Col-0, sid2, npr1 and sid2npr1 plants were infiltrated with 106 CFU/ml of either Pph or Pph (HopM1). Bacterial growth was assayed at 0, 2 and 4 days after infiltration. Graph represents the combined result from 5 different biological replicates for day 4 and 2 biological replicates for day 2. The dashed line represents bacterial levels at day 0. Error bars represent standard deviations. (TIF) [file pone.0082032.s003.tif]
